# A Spatial Multi-Omic Framework Identifies Gliomas Permissive to TIL Expansion

**DOI:** 10.21203/rs.3.rs-6314842/v1

**Published:** 2025-04-25

**Authors:** Mustafa Khasraw, Kelly Hotchkiss, Kenan Zhang, Anna Corcoran, Elizabeth Owens, Pamela Noldner, Chelsea Railton, Kyra Van Batavia, Ying Zhou, Jodie Jepson, Kirit Singh, Roger McLendon, Kristen Batich, Anoop Patel, Katayoun Ayasoufi, Michael Brown, Evan Calabrese, Jichun Xie, Jose Conejo-Garcia, Beth Shaz, John Hickey

**Affiliations:** Duke University Medical Center; Duke University Medical Center; Duke University Medical Center; Duke University Medical Center; Duke University Medical Center; Duke University Medical Center; Duke University Medical Center; Duke University Medical Center; Duke University Medical Center; Duke University Medical Center; Duke University; Duke University; Duke University Medical Center; Duke University; Duke University Medical Center; Duke University; Duke University Medical Center; Duke University; Duke School of Medicine; Duke University Medical Center; Duke University Medical Center

**Keywords:** Glioblastoma, Astrocytoma, Tumor-Infiltrating Lymphocytes, Immunotherapy, TCR Profiling, Spatial Transcriptomics, Multiplex Proteomics, Single-Cell RNA Sequencing, Tumor Microenvironment, TIL Expansion

## Abstract

Tumor-infiltrating lymphocyte (TIL) therapy, recently approved by the FDA for melanoma, is an emerging modality for cell-based immunotherapy. However, its application in immunologically “cold” tumors such as glioblastoma remains limited due to sparse T cell infiltration, antigenic heterogeneity, and a suppressive tumor microenvironment. To identify genomic and spatial determinants of TIL expandability, we performed integrated, multimodal profiling of high-grade gliomas using spectral flow cytometry, TCR sequencing, single-cell RNA-seq, Xenium *in situ* transcriptomics, and CODEX spatial proteomics.

Comparative analysis of TIL-generating (TIL^+^) versus non-generating (TIL^−^) tumors revealed that *IL7R*expression, structured perivascular immune clustering, and tumor-intrinsic metabolic programs such as *ACSS3* were associated with successful TIL expansion. In contrast, TIL^−^tumors were enriched for neuronal lineage signatures, immunosuppressive transcripts including *TOX* and *FERMT1*, and tumor-connected macrophages.

This study defines spatial and molecular correlates of TIL manufacturing success and establishes a genomics-enabled selection platform for adoptive T cell therapy. The profiling approach is now being prospectively implemented in the GIANT clinical trial (NCT06816927), supporting its translational relevance and scalability across glioblastoma and other immune-excluded cancers.

## Introduction

Tumor-infiltrating lymphocytes (TIL) therapy has shown efficacy in immune checkpoint inhibitor resistant melanoma^1,2^. The diverse tumor-specific targets of TIL make it an attractive therapy for heterogeneous tumors and resistance is less likely compared with cell therapies that have one or few targets^3^. In February 2024, the FDA approved lifileucel as the first TIL therapy to treat cancer confirming the future use of TILs in mainstream practice. However, the development of TIL therapy in immunologically “cold” tumors like high-grade gliomas is hampered by variability in TIL expansion, functional exhaustion of T cells, systemic and local immunosuppression, and tumor heterogeneity.

Prior studies highlight the role of T cell spatial heterogeneity and repertoire diversity in influencing TIL expansion potential^4^. Additionally, tumor-reactive TIL enrichment at baseline and clonotype dynamics have been identified as key determinants of therapeutic response, particularly in melanoma^5^. Integrated, multimodal characterization is emerging as a powerful approach for a holistic understanding of complex tumor microenvironments (TME) likely to drive future therapeutic precision approaches. Spatial omic technologies including genome, transcriptome, and proteome information retain spatial relationships to provide multidimensional, systems-level understanding of tumor biology^6–8^. Importantly, spatial approaches not only reveal the presence or absence of mutations, transcript abundance, and cell population compositions, but also offer insights into how spatial organization of cells and their coordinated cell-cell interactions drive differential responses to cancer treatment^9–14^. This may help characterize distinct immune multicellular environments across larger tissues^15^, identify networks of important cell-cell interactions, analyze how they change in different spatial niches^12^, track spatial and temporal tumor dynamics^11^, and identify potential prognostic and predictive molecular signatures^10^.

Despite using an optimized TIL expansion protocol, not all high-grade gliomas in our cohort successfully generated TILs. To investigate this variability, we performed an integrated multimodal analysis of cellular composition, TIL profiles, and molecular features. We conducted spectral flow cytometry on eighteen TIL products from eleven patients. We then focused on in-depth multi-omic comparisons of four tumors that generated yielding ≥10^8^ viable TILs and four non-generating tumors, all cultured under identical IL-2–based conditions. Expanded TILs were characterized via spectral flow cytometry and TCR sequencing to assess immune subsets, activation states, and clonality. Matched tumor tissues underwent single-cell RNA sequencing (scRNA-seq), *in situ* spatial transcriptomics (Xenium) with a custom glioma–immune gene panel and multiplexed spatial proteomics (CODEX) to evaluate immune infiltration and spatial architecture. Comparative TCR-seq and immunophenotyping revealed key differences in immune composition and clonal expansion. This systems-level analysis identifies features linked to successful TIL expansion, informing strategies to improve TIL manufacturing and immunotherapy efficacy in highgrade gliomas.

## Results

### Optimized IL-2–mediated TIL expansion protocol expands conventional CD4+ and CD8+ TILs

**Figure 1:**
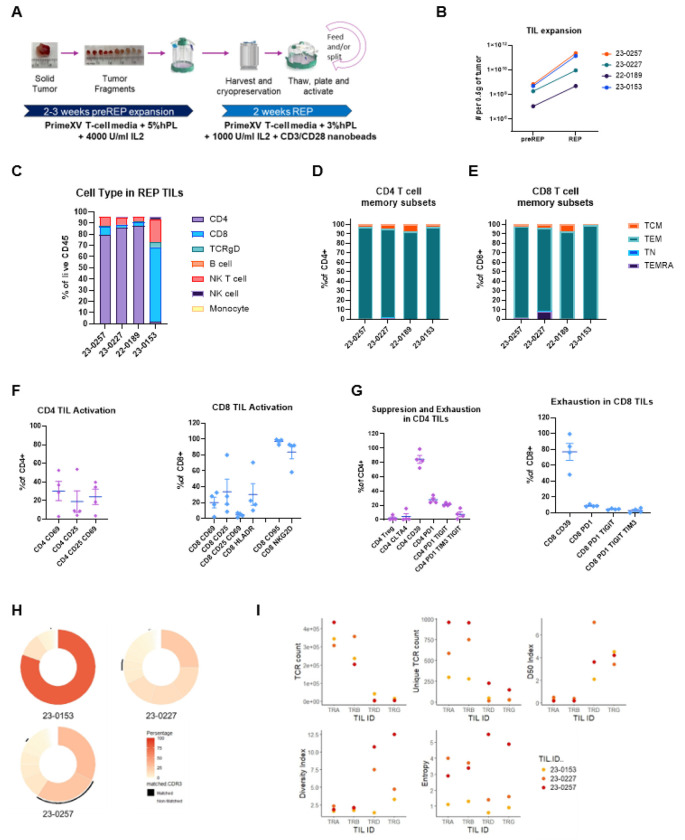
Pre-Rapid Expansion Protocol (preREP) and Rapid Expansion Protocol (REP) Tumor-Infiltrating Lymphocyte (TIL) T-Cell Receptor (TCR) and Flow Cytometry Analysis. (A) Schematic diagram of TIL expansion. (B) Expansion of TILs from preREP to REP with TIL numbers significantly increasing from preREP to REP (**paired t-test, p = 0.020**). Flow analysis characterizing (C) overall cell types and memory T cell subsets in (D) CD4 and (E) CD8 cells in REP TIL product from patients 23-0257, 23-0227, 22-0189, and 23-0153. (F) Percentage of activated CD4 and CD8 cells expressing CD69, CD25, HLA-DR as well as cytolytic molecules CD95 and NKG2D. (G) Suppressive CD4+ T-cell subsets (Tregs), and exhausted CD4 and CD8 cells. (H) Donut plots showing TCR clonotype distribution in TIL products, with black bands indicating the overlap between tumor-resident and expanded T-cell populations. (I) Dot plots comparing TIL expansion across key clonality metrics (TCR count, unique TCR count, D50 Index, diversity index, and entropy).

We attempted TIL expansion from glioblastoma and anaplastic astrocytoma tumors resected during surgery using an IL-2–based protocol (Fig. 1A). Final TIL yield depends on both pre-Rapid Expansion Protocol (pre-REP) numbers and Rapid Expansion Protocol (REP) fold expansion, both highly variable. We defined a minimum preREP threshold of 10^6^ TILs per 0.5g tumor to initiate REP, and a successful expansion as ≥10^8^ final TILs, sufficient for downstream analysis and potential therapeutic use. This threshold reflects prior studies showing that effective TIL therapy requires robust *ex vivo* expansion while preserving cell viability and function.

Of eighteen expanded TIL products, eleven were CD4^+^ dominant, three were CD8^+^ dominant, three were evenly split, and one was enriched for NK T cells (Supplementary Fig. 1). Four samples from individual patients met the ≥10^8^ TIL yield and were selected for in-depth analysis. All showed significant expansion from preREP to REP (100–1,000-fold increase, Fig. 1B), meeting criteria for successful TIL expansion. Expanded TILs consisted mainly of CD4^+^ and CD8^+^ T cells (Fig. 1C). In two patients analyzed across both phases, cell composition remained stable (Supplementary Fig. 1B–C). Most CD4^+^ and CD8^+^ TILs exhibited an effector memory phenotype (CD45RA^−^CCR7^−^; Fig. 1C, 1E).

CD4^+^ T cells included activated subsets: 30% CD69^+^ (early) and 25% CD25^+^ (late; Fig. 1F). CD8^+^ T cells had 20% CD69^+^, 35% CD25^+^, and 30% HLA-DR^+^, consistent with cytotoxic function. Although CD8^+^ percentages were low in some samples, >80% expressed cytolytic markers CD95 and NKG2D, indicating strong killing potential. We also assessed suppressive and exhausted populations. Regulatory T cells (CD4^+^CD25^+^Foxp3^+^) were <10% of CD4^+^ T cells, and CTLA4^+^ suppressive CD4^+^ cells were <20%. Thus, high CD4^+^ content did not reflect a high regulatory phenotype. Exhausted CD4^+^ and CD8^+^ T cells (PD1^+^TIGIT^+^TIM3^+^) were present at low levels and lacked LAG3, suggesting a potentially non-terminally exhausted state (Fig. 1G).

To assess clonal overlap, we performed TCRseq on TIL products and matched tumor tissues. Donut plots showed limited clonal overlap (Fig. 1H). Although γδ TCRs were rare relative to αβ TCRs, their detection in TIL^+^ products confirmed successful expansion (Fig. 1I), consistent with flow cytometry (Fig. 1C, Supplementary Fig. 1D). TCR metrics showed higher D50 and Diversity Index in γδ vs. αβ T cells, indicating a broader TCR repertoire and less clonal dominance in γδ T cells—suggesting a more diverse and potentially adaptable immune response.

### Successful TIL expansion associated with baseline clonal frequency, intratumoral inflammation, and lower Treg signatures

**Figure 2:**
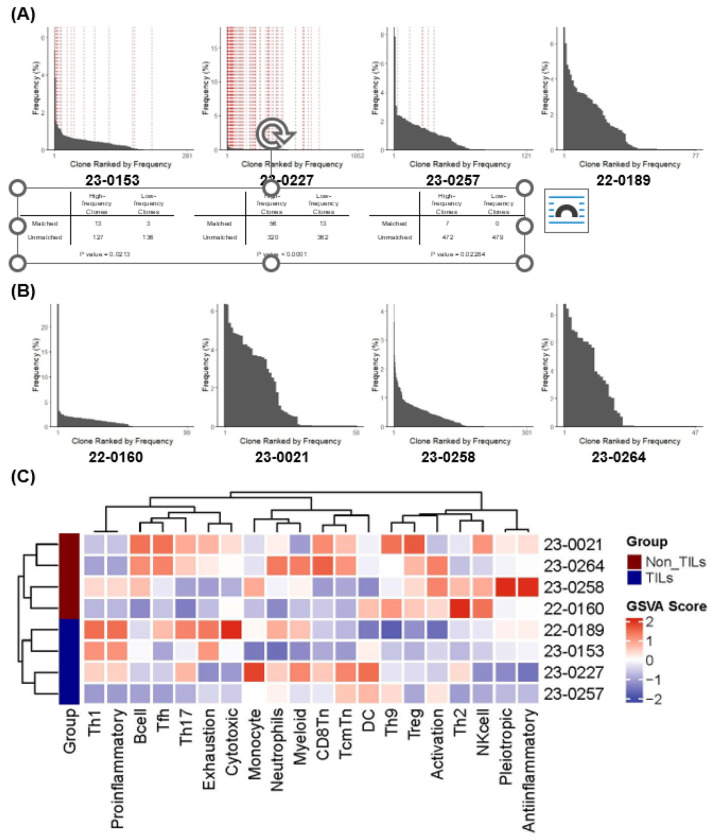
T-cell receptor (TCR) sequencing of Tumor-Infiltrating Lymphocyte (TIL) and corresponding tumor tissues. **(A)** Overlay histogram of TIL+ samples showing the clonal overlap between tumor-resident and expanded TIL populations. Histogram displaying the distribution of TCR clonotypes within the tumor tissue. Each bar represents the frequency of a unique TCR clone within the tumor-resident T-cell population, ranked from most frequent to the least. The **vertical red dashed lines** indicate shared T-cell clonotypes present in both the tumor tissue and the expanded TIL product. Higher overlap in the more frequent half of the TCR clones suggests that pre-existing tumor-reactive T cells were successfully propagated during expansion (Chi-squared test with Yates’ continuity correction). TCRseq of TIL product was not conducted in sample 22-0189 because of limited generation of TIL, hence no matched TCR clone was shown. **(B)**: Histogram of clonal distribution of TIL− tumor tissues. **(C)** Immune Pathway and Cell Type Enrichment: Heatmap of GSVA scores showing key immune pathways in TIL+ tumors (blue) and TIL− (red) tumors. TIL+ tumors exhibit higher Th1 and pro-inflammatory signatures, while TIL− tumors show increased Treg, Th2, and anti-inflammatory pathways. These data provide insight into TIL clonal dynamics and their potential contribution to anti-tumor immunity.

To compare clonal distribution, we performed TCRseq on tumor tissues from both TIL^+^ and TIL^−^ samples, along with non-malignant brain tissues from epilepsy surgery as controls (data not shown due to insufficient yield). While overall clonality did not differ significantly between TIL^+^ and TIL^−^ tumors, TIL^+^ samples showed higher total and unique CDR3 reads (Supplementary Fig. 2), indicating greater T cell abundance or a more diverse TCR repertoire. Parallel library construction in non-malignant tissues yielded insufficient material, consistent with low lymphocyte infiltration. TIL^+^ tumors also showed a trend toward a lower D50 index—meaning fewer T cell clones accounted for half of the total TCR repertoire—suggesting more selective clonal expansion, though the difference was not statistically significant. **T cell receptor beta (TRB) clone distributions**, representing the diversity and frequency of T cells based on their beta chain sequences, were ranked by frequency in TIL^+^ and TIL^−^ tumors (Fig. 2A–B). In three TIL^+^ cases with matched TIL product TCRseq, high-frequency clones in tumor were also detected in expanded TILs, suggesting clonal expansion during culture (Fig. 2A). Using a regulatory T cell (Treg) gene expression signature (Supplementary Table 2: FOXP3, IKZF2, IL10, LAG3, TGFB1, IL2RA, STAT5A), we performed immunophenotyping by next generation sequencing. TIL^+^ tumors showed significantly lower Treg signature expression than TIL^−^ tumors (p=0.029, Mann–Whitney U test), suggesting a more immune-permissive TME (Fig. 2C). Additionally, Th1 and pro-inflammatory gene signatures were enriched in TIL^+^ tumors, further highlighting immune landscape differences between TIL^+^ and TIL^−^ tumors.

#### Clinical and MRI imaging features of TIL+ vs. TIL− tumors

**Figure 3:**
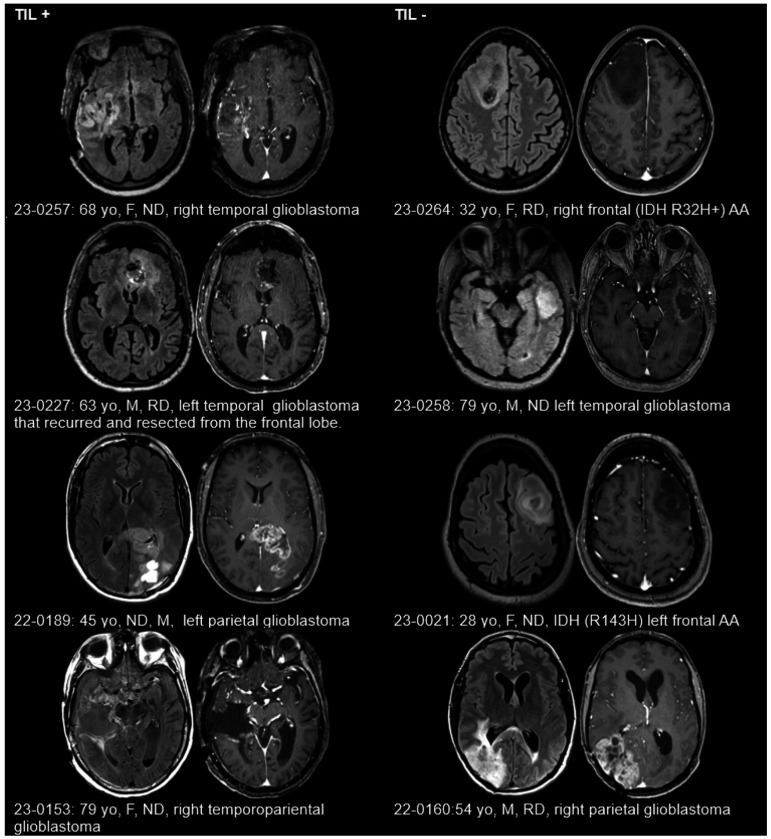
Clinical and MRI Characteristics of eight glioma patients arranged by Tumor-Infiltrating Lymphocyte (TIL) expansion success during *ex vivo* expansion with IL-2. For each patient, axial T2-weighted FLAIR (left) and T1-weighted post-contrast (right) images are presented, highlighting tumor location and imaging features. Abbreviations: F: Female, M: Male, R: Recurrent Disease, ND: Newly Diagnosed Disease. IDH: Isocitrate Dehydrogenase mutations: AA: Anaplastic Astrocytoma, yo: years old.

The eight patients with high-grade gliomas ranged from 28 to 79 years old (Fig. 3), with a mix of newly diagnosed and recurrent tumors across various anatomical locations (frontal, temporal, parietal) (Supplementary Table 6). All TIL+ tumors were Isocitrate Dehydrogenase (IDH) wild type glioblastoma whereas TIL− tumors consisted of two IDH wild type glioblastoma and two IDH-mutated anaplastic astrocytoma. MRI features (Fig. 3) were heterogenous across both groups without any clear differences in enhancement pattern or non-enhancing peritumoral signal abnormality. Due to the small sample size, these findings should be interpreted with caution and require validation in larger cohorts.

### Distinct immune and cellular landscapes define TIL+ and TIL− high-grade gliomas

**Figure 4:**
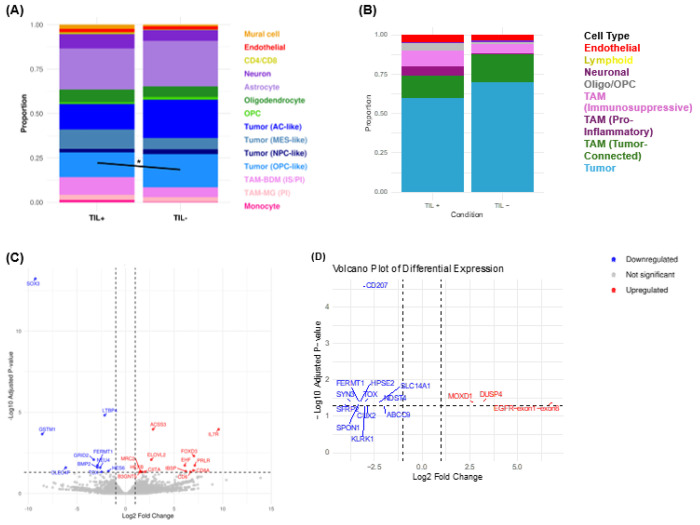
Immune Composition and Gene Expression Differences Between Tumor-Infiltrating Lymphocyte (TIL) + and – tumors **(A–B)** Immune Composition Analysis: Stacked bar plots depict the proportional differences in immune subsets between TIL+ and TIL− tumors. TIL+ tumors exhibit a higher presence of cytotoxic CD8+ T cells and pro-inflammatory immune subsets, while TIL− tumors are enriched in immunosuppressive populations, including Tregs and macrophages. **(C)**Volcano plot of differentially expressed genes between TIL+ and TIL− tumors. Genes upregulated in TIL+ tumors are highlighted in blue, while genes upregulated in TIL− tumors are shown in red. The x-axis represents the log2 fold change, and the y-axis represents the -log10 adjusted p-value. Genes with significant differential expression (adjusted p < 0.05) are annotated. **(D)**Volcano lot of differentially expressed genes in TIL+ and TIL− Tumors (Xenium Spatial Transcriptomics). TIL− tumors upregulate genes linked to tumor-intrinsic pathways and immune exclusion. These findings highlight the immune-permissive nature of TIL+ tumors, which contrasts with the immunosuppressive and immune-excluded phenotype of TIL− tumors.

To assess TME differences between TIL^+^ and TIL^−^ tumors, we performed scRNA-seq on FFPE tissue. TIL^+^ tumors were enriched in tumor-associated macrophages (TAMs), CD8^+^ T cells, and mesenchymal-like gene signatures, while TIL^−^ tumors had higher levels of oligodendrocyte precursor cells (OPCs), astrocytes, and neuronal lineage markers, features of an immune-excluded phenotype (Fig. 4). Proportional analysis confirmed significantly more OPC-like tumor cells in TIL^−^ tumors (p=0.03) and a trend toward increased glial and neuronal cells.

Differential gene expression (using adjusted p-values) revealed genes upregulated in TIL^+^ tumors (*ACSS3, IL7R, FOXD3, EHF, ELOVL2, IBSP*), suggesting a TME favorable for immune infiltration and TIL expansion (Fig. 4C). IL7R, a key regulator of T cell survival and proliferation—was among the top differentially expressed genes, supporting its role in cytokine-driven activation and TIL expansion. IL7R was most highly expressed in CD4^+^ and CD8^+^ T cells in TIL^+^ tumors but also present at lower levels in non-lymphoid cells (AC-, OPC-, MES-like, and astrocytic tumor cells). To clarify whether IL7R expression in T cells contributed to the TIL^+^ phenotype, we performed EdgeR-based differential analysis on CD4^+^/CD8^+^ cells alone, confirming IL7R upregulation in TIL^+^ tumors and linking it to T cell–intrinsic cytokine responsiveness and *ex vivo* expandability.

*ACSS3*, involved in mitochondrial acetate and propionate metabolism, was also significantly upregulated in TIL^+^ tumors and remained a top differentially expressed gene in AC-like cells, suggesting a tumor-intrinsic metabolic state promoting immune-permissive phenotypes, rather than shifts in cellular composition. In contrast, genes upregulated in TIL^−^ tumors (*LTBP4, GSTM1, FERMT1, GRID2, BMP2, TOX, HES6*) suggested TIL expansion limitation through effects on immune infiltration or T cell dysfunction. These genes are linked to TGF-β signaling, oxidative stress resistance, immune evasion, and exhaustion, factors contributing to TIL persistence and function.

Though not statistically significant, in the Xenium data, TIL^+^ tumors tended toward more lymphoid cells and pro-inflammatory TAMs, while TIL^−^ tumors had more tumor cells, OPCs, and immunosuppressive TAMs. Individual UMAPs showed distinct cellular profiles—greater immune infiltration and activation in TIL^+^ tumors vs. immune-excluded features in TIL^−^ tumors (Supplementary Fig. 3). Despite trends in immune composition, TCR clonality, and gene expression, the small sample size (n=8) limited statistical power. Larger cohorts and multi-region sampling, from necrotic core to proliferative edge—are needed to validate these findings and assess clinical significance.

### Tumor-connected TAMs: Product of phagocytosis or potential driver of immune exclusion?

**Figure 5:**
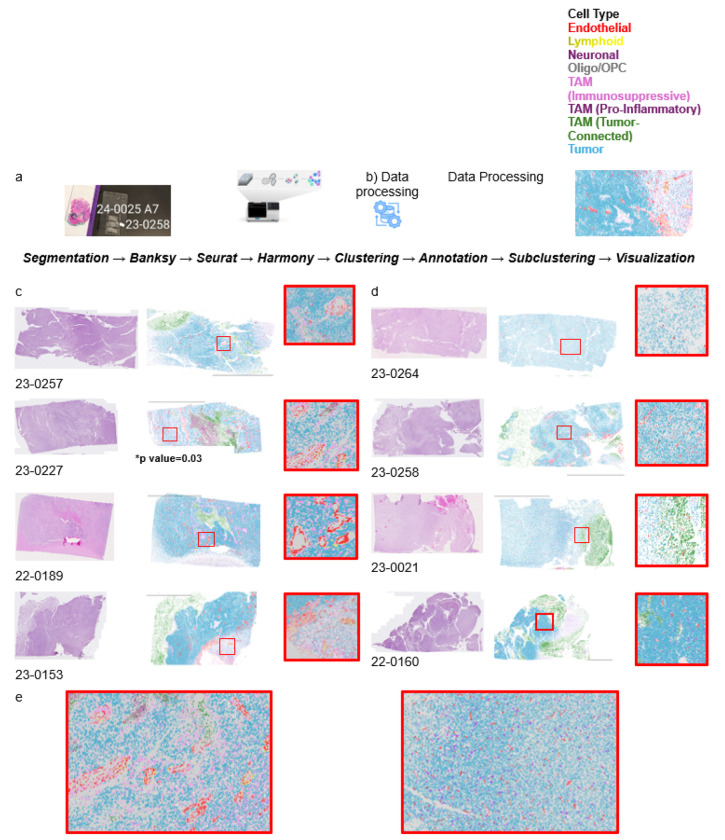
Histological and Spatial Transcriptomic Characterization of Tumor-Infiltrating Lymphocyte (TIL) + and − tumors. H&E-stained sections from TIL+ tumors illustrate tissue architecture and cellular density. Xenium spatial transcriptomic maps show gene expression distribution, while high-resolution Xenium Explorer images highlight lymphoid cells (yellow) near endothelial cells (red), suggesting an immune-permissive microenvironment. Spatial immune clustering, associated with successful TIL expansion, also identifies tumor-connected TAMs (green) localized in necrotic, fibrotic, or liquefied tumor regions. In contrast, TIL− tumors exhibit preserved architecture with sparse immune cell populations. Xenium Explorer images reveal tumor and stromal components of these immunologically “cold” tumors with minimal immune clustering. Scale bars represent 100 μm.

We identified a TAM subcluster expressing markers of tumor stemness *(SOX2, NES, MBP*), neural lineage (*PTPRZ1, NCAM1*), and glial identity (*AQP4, MBP, GJA1*). While this profile suggests a distinct TAM phenotype, alternative explanations include phagocytosis of tumor material, reactive astrocytes expressing macrophage genes, or microglia that have engulfed tumor cells. Unlike droplet-based scRNA-seq, where such signals may result from doublets, our probe-based spatial transcriptomics’ segmentation algorithm reduces artifact likelihood. Due to the unclear nature of these TAMs, we refer to them as tumor-connected TAMs (Fig. 4B, 5; Supplementary Fig. 3–4, 7). Though not statistically significant, they trended more abundant in TIL^−^ tumors, suggesting a possible link to immune exclusion and reduced T cell infiltration.

### Spatial immune clustering associates with successful TIL expansion

Spatial *in situ* transcriptomics imaging (Xenium) and multiplexed proteomics (CODEX) revealed distinct immune architectures in TIL^+^ vs. TIL^−^ tumors. In TIL^+^ tumors, CD8^+^ and CD4^+^ T cells formed structured clusters near vasculature, suggesting spatial immune surveillance and tumor–immune interaction (Fig. 5). In contrast, TIL^−^ tumors showed a diffuse, disorganized immune landscape with minimal T cell infiltration, indicative of an immune-excluded phenotype (Fig. 5). CODEX imaging confirmed perivascular immune aggregation in TIL^+^ tumors, absent in TIL^−^ samples (Fig. 5, 6C–D). Quantitative spatial analysis linked immune clustering in TIL^+^ tumors to increased CD163^+^ macrophages and higher CD8^+^ T cell density, suggesting a coordinated immune response within the glioma microvasculature.

## Discussion

High-grade gliomas, particularly glioblastoma, remain a major clinical challenge due to their immunosuppressive microenvironment and limited treatment options. As a prototypical “cold” tumor, glioblastoma raises critical questions about the feasibility of TIL therapy in overcoming immune barriers. Despite low baseline T cell infiltration, we optimized TIL expansion protocols and showed that TILs enriched in CD8^+^, conventional CD4^+^, and γδ T cells, with minimal regulatory T cells, can be reliably expanded from resected high-grade glioma using a modified IL-2 preREP followed by standard REP (Fig. 1 and supplementary Fig 1). However, not all tumors support expansion, underscoring the need to understand influencing factors. Molecular profiling revealed distinct immune landscapes in TIL^+^ vs. TIL^−^ tumors. TIL^+^ tumors showed pro-inflammatory clustering and immune activation, while TIL^−^ tumors were enriched in neuronal-like features and suppressive markers. Mesenchymal-like programs correlated with immune infiltration in TIL^+^ tumors, whereas OPC and astrocytic signatures predominated in TIL^−^ tumors. These findings suggest TIL expansion depends not only on lymphocyte presence but also on the functional and cellular state of the TME.

IL7R gene was upregulated in tumors with successful TIL expansion, suggesting that responsiveness to homeostatic cytokine signaling^16^ (e.g., IL-7) may support the maintenance and proliferative potential of T cells during *ex vivo* expansion. While IL-2 has traditionally been used to support TIL growth, its use can promote terminal differentiation and T cell exhaustion, ultimately compromising T cell persistence and anti-tumor functionality following infusion^17^. IL-7 also promotes a memory phenotype^18^, enhances Chimeric Antigen Receptor (CAR) T-Cell Therapy infiltration into solid tumors^19^, and as we have shown recently in murine glioma models^20^, increases CD8+ T cell numbers. Taken together, these findings highlight the need for future studies directly comparing IL-7– and IL-2–based TIL expansion protocols to determine whether IL-7 enhances effector function and reduces inhibitory signaling. Further investigation is warranted to validate *IL7R* as a predictive biomarker and to assess the potential of IL-7 as an alternative or adjunct to IL-2 in optimizing TIL expansion for therapeutic use.

In addition to well-established T cell-related genes, we identified upregulation of *ACSS3* and *GSTM1* in the TIL^+^ tumors, genes that may influence tumor and immune metabolism. Although speculative, *ACSS3*, which mediates acetate metabolism^21^, and supports lipid biosynthesis and metabolic flexibility in tumor cells, was upregulated in TIL^+^ **tumors**, including within **AC-like tumor cells**. This suggests a tumor-intrinsic metabolic adaptation that may promote a microenvironment favorable to TIL survival or expansion. These findings raise the possibility that *ACSS3* could function both as a biomarker and a mediator of TIL-supportive states. Whether it directly facilitates immune infiltration or enhances tumor resilience under immune pressure remains to be tested. *GSTM1*, an enzyme involved in detoxification and oxidative stress response, catalyzes glutathione conjugation for cellular elimination^21^. Its upregulation in TIL− tumors suggests a role in tumor adaptation to oxidative stress, potentially contributing to immune suppression. However, direct evidence of its impact on immune modulation in glioblastoma is lacking. Functional studies are needed to determine whether any of these genes plays an active role in shaping the tumor immune microenvironment or if its expression reflects broader metabolic reprogramming.

Pseudobulk RNA expression analysis using EdgeR on both Xenium and scRNA-seq data identified overlapping elevated expression of *TOX* and *FERMT1* in TIL^−^ tumors. This is notable given that scRNA-seq includes >18,000 genes, while our Xenium panel only included a customized 480-gene panel. Although the differential expression analysis does not account for spatial context in the Xenium data, the results still highlight key features of immune-permissive TMEs in TIL^+^ tumors (Fig. 4). Upregulation of *MOXD1* and *DUSP4* in the TIL^+^ tumors suggests a microenvironment conducive to immune infiltration and TIL persistence. *MOXD1* is involved in the development of glioblastoma^22^, but its functional role remains unclear and warrants further investigation. *DUSP4* activity affects cell migration^23^, proliferation^24^, extracellular matrix^23^. Together, upregulation of these genes in TIL+ tumors supports a niche in which TILs remain functionally active and capable of expansion.

*FERMT1* was consistently upregulated in TIL^−^ tumors across both scRNA-seq and Xenium pseudobulk DGE analyses, suggesting a role in promoting immune exclusion. *FERMT1* encodes kindlin-1, an integrin-activating protein highly expressed in epithelial tissues, originally linked to Kindler syndrome and implicated in various cancers^25,26^. *FERMT1* may help establish a physical and molecular barrier to T cell infiltration, supported by transcriptional signatures of increased tumor stemness; its suppression reduces stemness and promotes anti-tumor effects in glioma cells^27^, indicating a more undifferentiated and immune-resistant TME in TIL^−^ tumors. In contrast, TIL^+^ tumors showed elevated *IL7R* expression, indicating a more immune-permissive environment where *IL7R*-driven homeostatic signaling may support T cell survival and expansion.

Xenium and multiparametric flow cytometry identified distinct TAM subsets in TIL^−^ tumors. Immunosuppressive TAMs expressed CD68, CD163, *TGFB1, TREM2, and TMEM119*; pro-inflammatory TAMs expressed CXCL8, CXCR4, and CCL5. TIL^+^ tumors showed increased immune recruitment (CXCL8, CCL2, CD163), while TIL^−^ tumors expressed immunosuppressive markers (*TOX, SFRP2, LTBP4*) and neuronal genes (*NRXN1, OLIG2*), indicating tumor-driven immune evasion.

A TAM subcluster expressed tumor stemness genes (*SOX2, NES, MBP*), neural lineage markers (*PTPRZ1, NCAM1*), and glial markers (*AQP4, MBP, GJA1*), suggesting origins via phagocytosis, reprogramming, or mimicry. We designated these tumor-connected TAMs. H&E and Xenium analysis showed they localize to necrotic, edematous, or fibrotic regions, separated from viable tumor (Supplementary Fig. 7). This pattern was consistent across specimens and histologies. Though not statistically significant, they appeared to be more abundant in TIL^−^ tumors (Fig. 4B), potentially contributing to immune exclusion. Their role—passive or active—requires further study via lineage tracing and functional assays.

**Figure 6:**
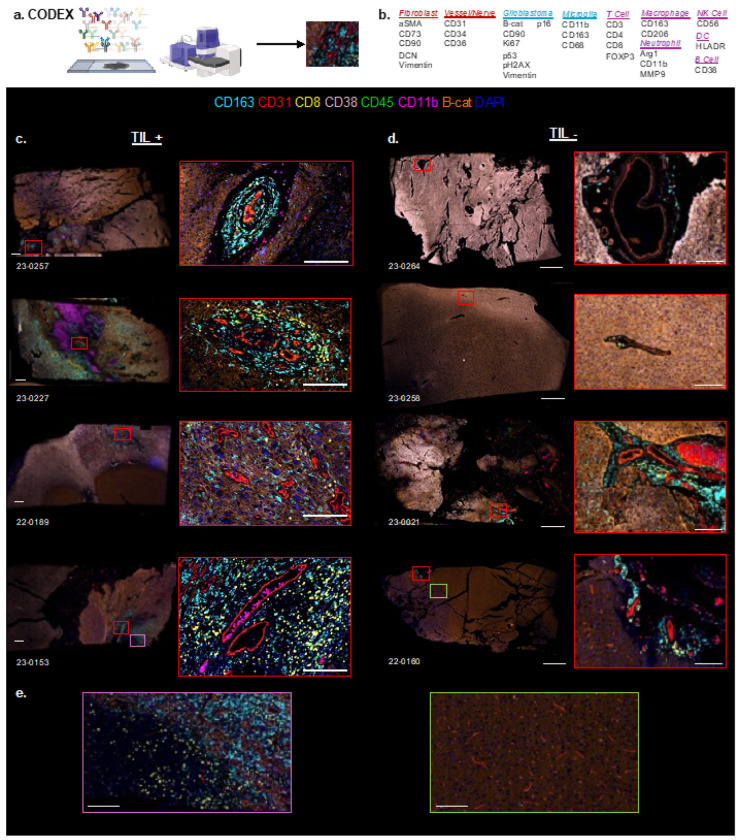
Co-Detection by Indexing (CODEX) analysis of Tumor-Infiltrating Lymphocyte (TIL) + and − tumors. (A) Schematic representation of multiplex staining and imaging workflow. (B) Marker panel includes CD163 (macrophages), CD31 (vasculature), CD8 (cytotoxic T cells), CD38 (activated immune cells), CD45 (leukocytes), CD11b (myeloid cells), B-catenin (tumor cells), and DAPI (nuclei). (C) Glioblastoma samples with successful TIL expansion (TIL+). Insets (right) highlight regions of immune infiltration, showing clustering of CD8+ T cells and other immune subsets. (D) Glioblastoma samples without TIL expansion (TIL−). Insets (right) reveal sparse immune cell presence with limited CD8+ T cell infiltration. (E) Comparison of TIL+ and TIL− samples, illustrating differential immune cell distribution and density. Scale bars represent 100 μm.

Beyond molecular profiling, spatial immune architecture distinguished TIL^+^ tumors, where lymphoid cells formed structured perivascular aggregates (Fig. 5–6), suggesting vascular niches support antigen presentation and T cell activation, consistent with single-cell studies showing endothelial and mural cell specialization in coordinating immune infiltration in glioblastoma^28^. In contrast, immune cells in TIL^−^ tumors were sparse and disorganized, with minimal intratumoral penetration (Fig. 5–6). This study also confirms the well-documented intra- and interpatient heterogeneity of glioblastoma^29^. Like findings in other studies^30,31^, immunosuppressive myeloid-tumor interactions within specific cell niches may not only inhibit anti-tumor responses but also prevent successful TIL generation. Tertiary lymphoid structures (TLS) have been described in gliomas^32,33^, but the immune aggregates observed here (Fig. 5) do not meet the criteria for mature TLS in cancers^34^, necessitating further characterization. Emerging studies also highlight the prognostic and predictive value of spatial data in gliomas^35,36^.

A persistent limitation across most spatial transcriptomic and single-cell RNA sequencing studies—including those published in high-impact journals—is their reliance on retrospectively banked biospecimens. While these studies have yielded important insights, they are inherently vulnerable to immortal time bias, as biospecimens are typically collected post hoc and outside defined clinical timelines. This bias constrains the ability to resolve temporal relationships between biological features and clinical outcomes, limiting the translational utility of candidate biomarkers.

To overcome these limitations, we prospectively integrated multi-platform immune and spatial profiling—including flow cytometry, TCRseq, scRNA-seq, Xenium *in situ* transcriptomics, and CODEX multiplexed spatial proteomics, within the Glioblastoma Immunotherapy Advancement with Nivolumab and Relatlimab (GIANT) trial (NCT06816927). This clinical trial enables real-time, pre-treatment tissue acquisition with longitudinal follow-up and uniformly annotated clinical metadata, establishing a rigorous framework for biomarker discovery and validation.

Despite the modest cohort size (n=8; 4 TIL^+^, 4 TIL^−^), convergence across orthogonal technologies supports the robustness of the findings. We observed consistent immunologic features across flow cytometry, TCR sequencing, single-cell and spatial transcriptomics, and multiplexed proteomics, enabling cross-platform validation. Where possible, we quantified overlap—for example, by calculating the percentage of TCRs shared between tumor tissues and expanded TIL products. Statistical analyses, including EdgeR for differential expression and Mann–Whitney U tests for group comparisons, were applied to maximize interpretability and analytical rigor.

This study identifies cellular and molecular features associated with the successful expansion of TILs from primary glioblastomas. I*L7R* expression and structured perivascular immune clustering were consistently enriched in TIL^+^ tumors, suggesting that local immune architecture may support effective TIL priming. Notably, tumor-intrinsic expression of *ACSS3*, a regulator of acetate metabolism—was upregulated in astrocyte-like malignant cells in TIL^+^ samples, suggesting a metabolic phenotype conducive to immune engagement. In contrast, TIL^−^ tumors were enriched for tumor-connected, immunosuppressive TAMs, consistent with a spatially immune-excluded phenotype.

These findings align with broader features of immune-cold tumors, including glioblastoma, pancreatic, prostate, and hormone receptor–positive breast cancers, where low antigenicity, MHC class I downregulation, and antigenic heterogeneity limit T cell engagement. Antigen specificity remains a major determinant of TIL efficacy, and approaches such as neoantigen-enriched TIL products, TCR-guided selection, or combination immunotherapies may improve outcomes in these settings. Our platform provides a mechanistic link between spatial immune context and antigen recognition, offering a path toward biomarker-driven patient stratification and personalized cell therapy manufacturing.

Importantly, this study should be interpreted as a discovery-stage, platform-development effort rather than a therapeutic efficacy evaluation. The integration of all profiling protocols within a prospective clinical trial framework addresses common limitations of retrospective datasets and establishes a foundation for future validation. Larger, multi-institutional cohorts with functional studies will be essential to confirm the predictive relevance of candidate biomarkers, including *IL7R*, spatial immune clustering, tumor-associated macrophage states, and tumor-intrinsic metabolic programs.

Together, these findings establish a spatially resolved, clinically integrated discovery platform to identify gliomas permissive to TIL therapy. The ability to couple real-time tissue acquisition with multimodal profiling and clinical annotation supports early decision-making on TIL manufacturing and informs the design of next-generation strategies, including neoantigen discovery pipelines and engineered TIL products. The approach is generalizable to other immune-resistant solid tumors, providing a translational framework to guide immunotherapy deployment in anatomically and immunologically challenging malignancies.

## Online Methods

### Sample Preparation

The glioma samples were collected as per Duke Institutional Review Board approved protocol “Collection of Solid Tumor Specimens for Pre-clinical Development and Manufacturing of a Clinical Tumor Infiltrating Lymphocyte (TIL) Cell-therapy Product” Pro00106078 for the collection of the preclinical development samples. Patients were consented for biobanking of their tumor and blood specimens, sample acquisition and data collection.

### TIL Expansion

Autologous TILs were produced in the process development lab at the Marcus Center for Cellular Cures at Duke University. TIL expansion was performed using a portion of the resected tumor that was distinct from the sample used for multimodality analyses, ensuring that immune profiling of the original tumor tissue remained unaffected by the *ex vivo* expansion process. Fresh tumors samples were weighted, cut to small fragments of 2-3mm^3^ and placed into G-Rex flasks at 5-24 mg/cm^2^ culture surface in preREP media containing PrimeXV T cell expansion media (FujiFilm), 5% human platelet lysate (hPL, Compass Biomedical), 4000 IU/ml IL-2 (Proleukin or BioTechne), Pen/Strep (Gibco) and expanded for 18-28 days. TILs were collected on harvest day, counted, cryopreserved in CryoStor CS10 (BioLife Solutions) and stored in N_2_. For REP expansion, preREP TILs were thawed, washed and placed into a G-Rex flask at 1e6/ml*cm^2^ in REP media (PrimeXV T cell expansion media, 3% hPL, 1000IU/ml IL-2) and activated by addition of CD3/CD28 nanoparticles (TransAct, Miltenyi Biotec). After 3 days, the activation was stopped by dilution with REP media to the fill mark of the G-Rex flask. Culture media was replaced when media lactate reached 15mM. REP TILs harvested on culture day 14 and cryopreserved in CS10.

In some cultures, additional immune-modulating antibodies were incorporated into the preREP phase to assess their impact on TIL expansion. These included atezolizumab (an anti–PD-L1 antibody), nivolumab and pembrolizumab (anti–PD-1 antibodies), and urelumab (an agonistic anti–4-1BB antibody). These agents were selected based on their known roles in enhancing T cell activation and survival by blocking inhibitory signals (PD-1/PD-L1 axis) or stimulating co-stimulatory pathways (4-1BB), and were added alongside IL-2 to test whether their inclusion could augment TIL yield or function.

### Flow Cytometry

Cryopreserved cells were rapidly thawed in a 37C water bath and resuspended in RPMI media with 2% fetal bovine serum. Cells were then pelleted at 300xg for 5 minutes at 4C and resuspended to count by hemocytometer. One million cells per well were loaded into a round bottom 96 well plate for flow staining. Cells were first stained with LiveDead Blue Fixable viability dye (Invitrogen), then blocked with buffer containing anti-CD16/32 (Invitrogen) and mono-block (BioLegend). A master mix containing all surface antibodies (Supplementary Table 1) was prepared in brilliant violet stain buffer (Invitrogen) and added on top of blocking buffer for 30 mins. Cells were then fixed and permeabilized using the eBioscience^™^ Foxp3 / Transcription Factor kit to allow for intracellular staining of FOXP3, CD69, CD137 and CTLA4. Spectral data was collected on the Cytek Aurora 5L and analyzed by FlowJo v10.

### Sample Preparation for multimodality analyses

Formalin-fixed parffin-embedded (FFPE) tissues were processed for single-cell and xenium profiling, followed by bulk TCRseq and CODEX multiplexed imaging.

#### Formalin-Fixed Paraffin-Embedded (FFPE)

5mm FFPE tissue sections are placed on a 10x Genomics Xenium slide. Tissue slides are deparaffinized and decrosslinked. Next, tissue is processed through Hybridization, Ligation & Amplification protocols. Prepared tissue slides are then loaded for imaging on the Xenium Analyzer for *in situ* analysis. Cyclical rounds of fluorescent probe hybridization, imaging, and removal generate optical patterns specific for each barcode, which are converted into a gene identity. Identified transcripts are then visualized using Xenium Explorer software. **H&E staining was performed on the slides following Xenium analysis to allow for histopathological assessment**.

#### FFPE with Custom Panel and Multimodal Segmentation

5um FFPE tissue sections are placed on a 10x Genomics Xenium slide and baked at 42C for 3hours. Once dried, tissue slides are stored in a desiccator to await processing. Xenium slides are then incubated at 60C for 2 hours and then deparaffinized and decrosslinked. Briefly, sections are deparaffinized via xylene immersion to solubilize the paraffin, ethanol to remove the solubilized paraffin and slowly hydrate the tissue, and then immersed in water to remove residual ethanol. Once hydrated, tissues slides are decrosslinked. Tissue slides are inserted into 10x Genomics Xenium slide cassettes. Decrosslinking buffer is then added to the well created by the cassette and incubated. After incubation, the buffer is removed, and the sample is washed. Tissue is then processed through Hybridization, Ligation & Amplification protocols. Briefly, a custom panel containing probes for 480 custom gene panel *(The gene list is available as a supplementary file)* enriched in genes relevant to glioma, neuronal and immune cells, is added to the tissue. The 480-gene panel was used to enable mapping of gene expression in the tissue architecture. Differential expression analyses highlighted immune-tumor dynamics in pro-inflammatory and immunosuppressive pathways. Each circularizable DNA probe contains two regions that hybridize to target RNA and a third region that encodes a gene-specific barcode. The two ends of the probes bind the target RNA and are ligated to generate a circular DNA probe. Following ligation, the circularized probe is amplified, producing multiple copies of the gene-specific barcode for each target. Tissue slides are then stained using the 10x Genomics Multimodal Cell Segmentation Kit. During Multimodal staining, antibodies used for cell segmentation bind their antigens in an overnight incubation, followed by post-incubation washes to remove excess antibodies. The cell segmentation stain is then enhanced by 10x Genomics proprietary reagents. The Multimodal Cell Segmentation kit stains for cell nuclei, membranes, and cell interior that are inputs for the 10x Genomics automated morphology-based cell segmentation analysis pipeline. Prepared tissue slides are then loaded for imaging on the Xenium Analyzer for *in situ* analysis. Fluorescently-labeled oligos bind to the amplified DNA probes. Cyclical rounds of fluorescent probe hybridization, imaging, and removal generate optical patterns specific for each barcode, which are converted into a gene identity. Identified transcripts are then visualized using Xenium Explorer software.

### Chromium Single Cell Gene Expression Flex (scFFPE-seq)

For each sample, 2x 25 μm FFPE curls were dissociated in accordance with 10x Genomics “Isolation of Cells from FFPE Tissue Sections for Chromium Fixed RNA Profiling protocol (User guide CG000632, Rev. B). Following cell isolation, samples underwent probe hybridization with one probe barcode per sample from the 10x Genomics Single Cell Fixed RNA Human Transcriptome Probe Kit. Approximately 151,050 cells per sample were pooled, washed, filtered, and loaded into 10x Genomics’ Next Gem Chip Q targeting a recovery of 8,000 cells per probe barcode. Sequencing libraries were generated following 10x Genomics’ User Guide CG000527 for Fixed RNA Profiling (Rev. E). The tumor samples from eight patients were used to generate two libraries following 10x Genomics’ User Guide CG000527 for Fixed RNA Profiling (Rev. E). These libraries were sequenced on an Illumina NovaSeq platform, resulting in four FASTQ files per library. Individual samples in each library were demultiplexed by their incorporated barcodes using CellRanger’s (v7.0.1) multi pipeline and the GRCh38-2020-A reference (10x Genomics). Following demultiplexing, sequencing data from each sample was obtained, enabling data analysis across both libraries. CellRanger’s aggr pipeline was utilized to aggregate data from the same patients, treating data from different libraries as independent replicates to evaluate any biological variability and to provide more substantial cell counts.

### Single-Cell RNA Sequencing (scRNA-seq)

CellRanger’s pipelines corrected for empty droplets, cells with few transcripts, an important variable in quality control filtering. Cells were further filtered to ensure high quality reads, which including removing ambient RNA and potential doublets. Specifically, cells with greater than 10000 detected transcripts, greater than 7000 detected genes, a mitochondrial gene proportion exceeding 20%, a cell complexity below 80% (log10Gene/log10UMI), and a hemoglobin gene proportion above 2% were excluded before downstream analysis. The hemoglobin gene threshold of 2% was stringent since only a few cells across the eight samples exceeded this level, reflecting the solid tissue origin of the samples. Genes present in fewer than three cells per sample were also removed to eliminate low-abundance genes and correct potential misreads from the reference transcript. Although a threshold of 30 cells is commonly used, this was adjusted due to the low cell count of these samples, ranging from 539 to 1429 cells. Ribosomal gene proportions, another common parameter, were not applicable because CellRanger’s reference transcriptome excludes ribosomal reads. Violin plots confirmed that filtering did not result in significant data loss, but this filtering ensures that downstream analysis will not be influenced by technical variations or losses.

After normalization and scaling, the data was batch-corrected and integrated using Seurat’s integration pipeline. The IntegrateLayers function employs canonical correlation analysis (CCA), a method akin to principal component analysis, to identify shared sources of variation between sample groups (TIL+ vs. TIL−). Jackstraw plots and clustree were used to determine the appropriate number of principal components and value of resolution, respectively, before running this analysis. This approach removes non-biological variation, accounts for confounding factors, and enables comparative analysis between groups. To ensure reproducibility, a random seed was set for Seurat’s RunPCA, FindClusters, and RunUMAP.

Post-integration, clusters identified using Seurat’s integration method were automatically annotated using the SingleR package (https://github.com/dviraran/SingleR?tab=readme-ov-file), referencing a single-cell RNA sequencing glioblastoma dataset from CELLxGENE (https://cellxgene.cziscience.com/collections/999f2a15-3d7e-440b-96ae-2c806799c08c). SingleR, a correlation-based method, matches the full transcriptome of query cells to reference cells rather than relying solely on marker gene expression. Annotation accuracy was assessed by identifying differentially expressed genes (DEGs) for each cluster using Seurat’s FindMarkers function, comparing each cluster against the remaining cells. Tumor subtypes were validated based on prior literature^37^, with AC-like clusters expressing GFAP, BCAN, SPARC, and DBI; MES-like clusters expressing HILPDA, CA9, ADM, and VIM; NPC-like clusters expressing DCX, DLX5, SOX4, DBN1, and RND3; and OPC-like clusters expressing NEU4, PTPRZ1, LIMA, VCAN, and OLIG1. Non-malignant clusters were further validated using marker genes from CellMarker2.0^38^ and published literature^39,40^: astrocytes (GFAP, CLU, AQP4, S100B), CD4/CD8 T cells (CD3E, CD3D, CD3G, GMZA, SKAP1), endothelial cells (VWF, CLDN5, CLEC14A, CDH5), mural cells (CD248, PDGFRB, RGS5, FOXF2), neurons (SYT1, SNAP25, GABRA1, NRCAM), oligodendrocytes (MOG, MBP, OLIG1, OLIG2, CLDN1), OPCs (SOX10, PDGFRA, APOD, OLIG1, OLIG2), TAM-BDM (CD163, CD14, TGFBI, ITGA4, MARCO), TAM-MG (TMIGD3, APOC2, SCIN, P2RY12, NAV3), and monocytes (LYZ, AREG, CD163, CD68, MXD1).

Differential gene expression (DEG) analysis was conducted between TIL+ and TIL− groups using EdgeR, a Bioconductor software package for examining differential expression of replicated count data^41^. These methods were also applied to identify differentially expressed genes among cell types within each group.

Cell composition analysis was performed between the two groups to identify the proportion of each cell type in the TIL+ vs TIL− samples. The Mann-Whitney U test was used to determine whether the differences in cell composition between the sample groups were significant. Notably, the proportion of OPC-like tumor cells was significantly higher in TIL− samples compared to TIL+ samples. Violin plots were also made to illustrate sample variability in cell compositions.

### Spatial Transcriptomics

For analysis of each Xenium sample, centroid (location), segmentation (cell boundaries), and the counts matrix data were extracted. Samples were loaded and filtered to keep cells with more than five features and more than ten counts, with 0.7% of cells removed on average. The *ComputeBanksy* (Prabhakar lab) function was used on all the samples with a lambda of 0.2 to create a spatially informed matrix. Both the regular expression and Banksy matrices were extracted along with cell location to create a Seurat object. The standard Seurat and Harmony pipeline (functions: *NormalizeData, FindVariableFeatures, ScaleData, RunPCA, FindNeighbors, FindClusters, RunUMAP*) (https://satijalab.org/seurat/articles/seurat5_integration) was used to integrate the eight samples with the *IntegrateLayers* function (method=HarmonyIntegration), and all further analysis was based on the harmony clusters. This produced eight clusters, which were manually labeled as: Glial/Tumor cells (clusters 0,1, 3, 7, based on GFAP, MKI67, OLIG2, SOX2, EGFR, CDKN2A, OLIG1, and TP53), TAMs (clusters 2 and 4, based on CD68, CD163, CXCL8, ITGAX, and CCL5), Lymphoid (cluster 8, based on PTPRC, CD3E, CD4, and CD8A), Oligodendrocyte/OPC (cluster 5, based on MAG, MBP, and SOX10), and Endothelial (cluster 6, based on VWF, PDGFB, and CD34). These were then further clustered based on the Banksy matrix for those cells to get more specific cell types. The same Seurat pipeline was run on the Banksy matrix for each group of cells, and then FindAllMarkers was run on the Harmony matrix for annotation. First, clusters 2 and 4 were extracted and reclustered, producing three clusters which were labeled as “Tumor-Connected TAM” (SOX2, NES, and MBP), “Immunosuppressive TAM” (CD68, CD163, TGFB1, TREM2, and TMEM119), and “Pro-inflammatory TAM” (CXCL8, CXCR4, and CCL5) based on their differentially expressed marker genes calculated by FindAllMarkers.

Then, the same steps were followed for clusters 0, 1, 3, 5, and 7 to get more accurate tumor and normal brain clusters, generating a total of eight new clusters. These were labeled as OPC-like (OLIG1, PDGFRA, NLGN3, and LHFPL3), MES-like (CD44, ANXA1, SOD2, VIM, and DDIT3), NPC-like (DCX, SAOX11, SOX4, and ASCL1), AC-like (GFAP, AQP4, and HOPX), Proliferative-1 (PCNA, FOXM1, and OLIG1), Proliferative-2 (PCNA, GOXM1, CCNB2, SOX11, ASCL1, and DCX), Oligo/OPC (OLIG1 and SERINC5), and Neuronal cells (SLC17A7, NRXN1, SYNPR, and GAD1) respectively, based on their FindAllMarkers gene results. Original cluster labels were compared to these new labels, and non-tumor clusters retained the majority of the cells, improving the validity of the clustering. All these subclusters were mapped back onto the original Harmony UMAP and then plots were made by TIL type (TIL or no TIL) and by each sample.

Differential gene expression (DEG) analysis was conducted between TIL+ and TIL− groups using EdgeR^41^. These methods were also applied to identify differentially expressed genes among cell types within each group. Statistical testing using Mann-Whitney was performed to determine whether the differences in cell composition between the sample groups were significant, and no significant differences were found.

### CODEX Multiplexed Imaging and Data

#### Array creation

Imaging data was collected from multiple brain samples from multiple donors on one slide. We included up to six tissues that were cut onto the same slide with a 4 μm section thickness and mounted onto Superfrost PLUS slides for further processing.

#### Antibody conjugation

Each antibody was conjugated to a unique oligonucleotide barcode using our previously established protocol^42^, after which the tissues were stained with the antibody–oligonucleotide conjugates and we validated that the staining patterns matched the expected patterns already established for immunohistochemistry within positive control tissues of the intestine or tonsil. Otherwise, expected co-expression of markers in the case of staining of suspension cells was taken into account for validating separate cell type and phenotype antibodies. First, antibody–oligonucleotide conjugates were tested in low-plex fluorescence assays and the signal-to-noise ratio was also evaluated at this step, then they were tested all together in a single CODEX multicycle. Antibody information can be found in Supplementary Table 1.

#### CODEX staining and imaging

The tissue arrays were stained with the validated panels of CODEX antibodies and imaged. Briefly, this entailed cyclic stripping, annealing, and imaging of fluorescently labeled oligonucleotides complementary to the oligonucleotide conjugated to the antibody as described^42^. Each slide underwent CODEX multiplexed imaging using Akoya Phenocycler Fusion machine; metadata from each CODEX run can be found in the supplementary files.

### TCR Repertoire and Immunophenotyping Analyses

T cell receptor sequencing (TCRseq) was performed using iRepertoire’s RepSeq+ technology to assess the diversity and clonality of immune repertoires. The diversity and clonality of tumor-infiltrating lymphocyte (TIL) populations were evaluated, with a specific focus on clonal expansions, particularly among CD8+ T cells in TIL+ tumors, to quantify tumor-reactive TCR frequencies. ImmunoSight, a targeted NGS assay of 150+ immunophenotype genes, alongside the RepSeq+ was performed to detect the immune response of the corresponding samples.

#### TCRseq

RNA was extracted from TIL samples using the PAXgene RNA Extraction Kit (PreAnalytix), and total RNA were extracted from FFPE samples using the Covaris truXTRAC FFPE total NA Plus Kit, and SPRIselect beads were used for RNA concentration to maximize input for amplification. 1600 ng of RNA per sample, along with replicates, was utilized for RepSeq+ TCR alpha, beta, delta, and gamma chain amplification. The RepSeq+ protocol facilitated simultaneous amplification of all four TCR chains under uniform conditions using primer pairs specific to V-D-J combinations, incorporating unique molecular identifiers (UMIs) during reverse transcription to minimize sequencing errors and PCR duplicates. Sequencing was performed on the Illumina NextSeq 1000 platform using a P1 600-cycle kit. Data analysis was conducted using the iRmap pipeline, which involved de-multiplexing sequence reads, mapping them to IMGT germline reference sequences, and extracting CDR3 regions. The final dataset was refined by collapsing identical CDR3 and UMI combinations to correct for sequencing errors. TCR repertoire diversity and clonality were evaluated by D50, Diversity index, and Shannon entropy. The D50 is the percentage of dominant and unique T cell clones that account for the cumulative 50% of the total CDR3s counted. The diversity index is 100 minus the area under the curve between the percentage of total reads and the percentage of unique CDR3s.

#### Immunophenotyping

A total of 5 μL of extracted RNA from both the TIL and FFPE samples was used for immunophenotyping. Library construction included cDNA purification via SPRIselect bead purification, followed by two rounds of gene-specific amplification and adapter ligation to generate Illumina-compatible libraries.

Immunophenotyping data were analyzed by aligning reads to a reference database of expected amplicons using bowtie2 with gene counts normalized across samples. Immune cell type and immune response scores were generated by Gene Set Variation Analysis (GSVA) and visualized by ComplexHeatmap. The gene signatures used are listed in Supplementary Table 2.

## Data Availability

Raw and processed data available on request.

## References

[1] Rohaan M.W. Tumor-Infiltrating Lymphocyte Therapy or Ipilimumab in Advanced Melanoma. New England Journal of Medicine 387, 2113–2125 (2022). 36477031 10.1056/NEJMoa2210233

[2] Sarnaik A.A. Lifileucel, a Tumor-Infiltrating Lymphocyte Therapy, in Metastatic Melanoma. J Clin Oncol 39, 2656–2666 (2021). 33979178 10.1200/JCO.21.00612 PMC8376325

[3] Creelan B.C. Tumor-infiltrating lymphocyte treatment for anti-PD-1-resistant metastatic lung cancer: a phase 1 trial. Nature medicine 27, 1410–1418 (2021). 10.1038/s41591-021-01462-y PMC8509078 34385708

[4] Poschke I.C. The Outcome of Ex Vivo TIL Expansion Is Highly Influenced by Spatial Heterogeneity of the Tumor T-Cell Repertoire and Differences in Intrinsic In Vitro Growth Capacity between T-Cell Clones. Clin Cancer Res 26, 4289–4301 (2020). 32303540 10.1158/1078-0432.CCR-19-3845

[5] Chiffelle J. Tumor-reactive T cell clonotype dynamics underlying clinical response to TIL therapy in melanoma. Immunity 57, 2466–2482.e12 (2024). 39276771 10.1016/j.immuni.2024.08.014

[6] Hickey J.W. Spatial mapping of protein composition and tissue organization: a primer for multiplexed antibody-based imaging. Nature Methods 19, 284–295 (2022). 34811556 10.1038/s41592-021-01316-y PMC9264278

[7] Bressan D., Battistoni G. & Hannon G.J. The dawn of spatial omics. Science 381, eabq4964 (2023). 37535749 10.1126/science.abq4964 PMC7614974

[8] Liu L. Spatiotemporal omics for biology and medicine. Cell 187, 4488–4519 (2024). 39178830 10.1016/j.cell.2024.07.040

[9] Chen J., Larsson L., Swarbrick A. & Lundeberg J. Spatial landscapes of cancers: insights and opportunities. Nature Reviews Clinical Oncology 21, 660–674 (2024). 10.1038/s41571-024-00926-7 39043872

[10] Matusiak M. Spatially Segregated Macrophage Populations Predict Distinct Outcomes in Colon Cancer. Cancer Discovery 14, 1418–1439 (2024). 38552005 10.1158/2159-8290.CD-23-1300 PMC11294822

[11] Hickey J.W. T cell-mediated curation and restructuring of tumor tissue coordinates an effective immune response. Cell Reports 42, 113494 (2023). 38085642 10.1016/j.celrep.2023.113494 PMC10765317

[12] Strasser M.K. Concerted epithelial and stromal changes during progression of Barrett’s Esophagus to invasive adenocarcinoma exposed by multi-scale, multi-omics analysis. bioRxiv, 2023.06.08.544265 (2023).

[13] Baertsch M.-A., Nolan G.P. & Hickey J.W. Multicellular modules as clinical diagnostic and therapeutic targets. Trends in Cancer 8, 164–173 (2022). 34872889 10.1016/j.trecan.2021.11.004 PMC8854341

[14] Hickey J.W. Integrating multiplexed imaging and multiscale modeling identifies tumor phenotype conversion as a critical component of therapeutic T cell efficacy. Cell Systems 15, 322–338.e5 (2024). 38636457 10.1016/j.cels.2024.03.004 PMC11030795

[15] Hickey J.W. Organization of the human intestine at single-cell resolution. Nature 619, 572–584 (2023). 37468586 10.1038/s41586-023-05915-x PMC10356619

[16] Yang S., Archer G.E., Flores C.E., Mitchell D.A. & Sampson J.H. A cytokine cocktail directly modulates the phenotype of DC-enriched anti-tumor T cells to convey potent anti-tumor activities in a murine model. Cancer immunology, immunotherapy: CII 62, 1649–1662 (2013). 10.1007/s00262-013-1464-0 PMC3850168 23982483

[17] Ross S.H. & Cantrell D.A. Signaling and Function of Interleukin-2 in T Lymphocytes. Annu Rev Immunol 36, 411–433 (2018). 29677473 10.1146/annurev-immunol-042617-053352 PMC6472684

[18] Chen D., Tang T.X., Deng H., Yang X.P. & Tang Z.H. Interleukin-7 Biology and Its Effects on Immune Cells: Mediator of Generation, Differentiation, Survival, and Homeostasis. Front Immunol 12, 747324 (2021). 34925323 10.3389/fimmu.2021.747324 PMC8674869

[19] ElKassar N. & Gress R.E. An overview of IL-7 biology and its use in immunotherapy. J Immunotoxicol 7, 1–7 (2010). 20017587 10.3109/15476910903453296 PMC2826542

[20] Pang N. IL-7 and CCL19-secreting CAR-T cell therapy for tumors with positive glypican-3 or mesothelin. J Hematol Oncol 14, 118 (2021). 34325726 10.1186/s13045-021-01128-9 PMC8323212

[21] Guertin D.A. & Wellen K.E. Acetyl-CoA metabolism in cancer. Nature Reviews Cancer 23, 156–172 (2023). 36658431 10.1038/s41568-022-00543-5 PMC11137663

[22] Shi P. MOXD1 knockdown suppresses the proliferation and tumor growth of glioblastoma cells via ER stress-inducing apoptosis. Cell Death Discovery 8, 174 (2022). 35393406 10.1038/s41420-022-00976-9 PMC8991257

[23] Paumelle R. Sequential activation of ERK and repression of JNK by scatter factor/hepatocyte growth factor in madin-darby canine kidney epithelial cells. Molecular biology of the cell 11, 3751–3763 (2000). 11071904 10.1091/mbc.11.11.3751 PMC15034

[24] Lin H., Qiu S., Xie L., Liu C. & Sun S. Nimbolide suppresses non-small cell lung cancer cell invasion and migration via manipulation of DUSP4 expression and ERK1/2 signaling. Biomedicine & Pharmacotherapy 92, 340–346 (2017). 28554129 10.1016/j.biopha.2017.05.072

[25] Liu C. FERMT1 mediates epithelial–mesenchymal transition to promote colon cancer metastasis via modulation of β-catenin transcriptional activity. Oncogene 36, 1779–1792 (2017). 27641329 10.1038/onc.2016.339

[26] Sin S. Role of the focal adhesion protein kindlin-1 in breast cancer growth and lung metastasis. Journal of the National Cancer Institute 103, 1323–1337 (2011). 21832234 10.1093/jnci/djr290

[27] Pan Z. FERMT1 suppression induces anti-tumor effects and reduces stemness in glioma cancer cells. Journal of Cancer Research and Clinical Oncology 150, 338 (2024). 38976072 10.1007/s00432-024-05859-3 PMC11231014

[28] Bejarano L. Single-cell atlas of endothelial and mural cells across primary and metastatic brain tumors. Immunity. 10.1016/j.immuni.2025.02.022 40107274

[29] Moffet J.J.D. Spatial architecture of high-grade glioma reveals tumor heterogeneity within distinct domains. Neuro-Oncology Advances 5(2023). 10.1093/noajnl/vdad142 PMC10699851 38077210

[30] Ravi V.M. Spatially resolved multi-omics deciphers bidirectional tumor-host interdependence in glioblastoma. Cancer Cell 40, 639–655.e13 (2022). 35700707 10.1016/j.ccell.2022.05.009

[31] Liu M. Spatial transcriptomics reveals segregation of tumor cell states in glioblastoma and marked immunosuppression within the perinecrotic niche. Acta Neuropathologica Communications 12, 64 (2024). 38650010 10.1186/s40478-024-01769-0 PMC11036705

[32] van Hooren L. Agonistic CD40 therapy induces tertiary lymphoid structures but impairs responses to checkpoint blockade in glioma. Nat Commun 12, 4127 (2021). 34226552 10.1038/s41467-021-24347-7 PMC8257767

[33] Shen S. Toll-like receptor agonists promote the formation of tertiary lymphoid structure and improve anti-glioma immunity. Neuro-Oncology 27, 140–154 (2024). 10.1093/neuonc/noae167 PMC11726345 39188155

[34] Vanhersecke L. Standardized Pathology Screening of Mature Tertiary Lymphoid Structures in Cancers. Laboratory Investigation 103, 100063 (2023). 36801637 10.1016/j.labinv.2023.100063

[35] Zheng Y., Carrillo-Perez F., Pizurica M., Heiland D.H. & Gevaert O. Spatial cellular architecture predicts prognosis in glioblastoma. Nature Communications 14, 4122 (2023). 10.1038/s41467-023-39933-0 PMC10336135 37433817

[36] Liu Y. Integration analysis of single-cell and spatial transcriptomics reveal the cellular heterogeneity landscape in glioblastoma and establish a polygenic risk model. Frontiers in Oncology 13(2023). 10.3389/fonc.2023.1109037 PMC10308022 37397378

[37] Neftel C. An Integrative Model of Cellular States, Plasticity, and Genetics for Glioblastoma. Cell 178, 835–849.e21 (2019). 31327527 10.1016/j.cell.2019.06.024 PMC6703186

[38] Hu C. CellMarker 2.0: an updated database of manually curated cell markers in human/mouse and web tools based on scRNA-seq data. Nucleic Acids Research 51, D870–D876 (2022). 10.1093/nar/gkac947 PMC9825416 36300619

[39] Farmen K. Monocyte markers correlate with immune and neuronal brain changes in REM sleep behavior disorder. Proc Natl Acad Sci U S A 118 (2021). 10.1073/pnas.2020858118 PMC7958435 33658371

[40] Chen Y. Tumor-associated monocytes promote mesenchymal transformation through EGFR signaling in glioma. Cell Rep Med 4, 101177 (2023). 37652019 10.1016/j.xcrm.2023.101177 PMC10518634

[41] Robinson M.D., McCarthy D.J. & Smyth G.K. edgeR: a Bioconductor package for differential expression analysis of digital gene expression data. Bioinformatics 26, 139–140 (2009). 19910308 10.1093/bioinformatics/btp616 PMC2796818

[42] Black S. CODEX multiplexed tissue imaging with DNA-conjugated antibodies. Nature Protocols 16, 3802–3835 (2021). 34215862 10.1038/s41596-021-00556-8 PMC8647621

